# Functional imaging-guided carbon ion irradiation with simultaneous integrated boost for localized prostate cancer: study protocol for a phase II randomized controlled clinical trial

**DOI:** 10.1186/s13063-022-06798-5

**Published:** 2022-11-08

**Authors:** Wei Hu, Ping Li, Zhengshan Hong, Xiaomao Guo, Yulei Pei, Zhenshan Zhang, Qing Zhang

**Affiliations:** 1 Department of Radiation Oncology, Shanghai Proton and Heavy Ion Center, Fudan University Cancer Hospital, Shanghai, 201321 China; 2 Shanghai Key Laboratory of Radiation Oncology (20dz2261000), Shanghai, China; 3 Shanghai Engineering Research Center of Proton and Heavy Ion Radiation Therapy, Shanghai, 201321 China; 4 Department of Radiation Oncology, Shanghai Proton and Heavy Ion Center, Shanghai, 201321 China; 5 Department of Research and Development, Shanghai Proton and Heavy Ion Center, Fudan University Cancer Hospital, Shanghai, 201321 China

**Keywords:** Prostate cancer, Carbon ion radiotherapy, Simultaneous integrated boost, PSMA PET/CT, mpMRI

## Abstract

**Background:**

Due to the physical dose distribution characteristic of “Bragg peak” and the biological effect as a kind of high linear energy transfer ray, heavy ion therapy has advantages over conventional photon therapy in both efficacy and safety. Based on the evidence that prostate cancer lesions before treatment are the most common sites of tumor residual or recurrence after treatment, simultaneous integrated boost radiation therapy for prostate cancer has been proven to have the advantage of improving efficacy without increasing toxicities.

**Methods:**

This study is a prospective phase II randomized controlled clinical trial evaluating the efficacy and safety of functional imaging-guided carbon ion irradiation with simultaneous integrated boost for localized prostate cancer. One hundred and forty patients with localized prostate cancer will be randomized into carbon ion radiotherapy group and simultaneous integrated boost carbon ion radiotherapy group at a 1:1 ratio. The primary endpoint is to compare the incidence of treatment-related grade 2 and higher acute toxicities between the two groups according to National Cancer Institute Common Terminology Criteria for Adverse Events (NCI-CTCAE) version 4.03. Secondary endpoints are late toxicities, biochemical relapse-free survival, overall survival, progression-free survival, and quality of life.

**Discussion:**

This study adopts functional imaging-guided simultaneous integrated boost of carbon ion radiotherapy for localized prostate cancer, aiming to evaluate the differences in the severity and incidence of acute toxicities in patients with localized prostate cancer treated with carbon ion radiotherapy and simultaneous integrated boost carbon ion radiotherapy, in order to optimize the carbon ion treatment strategy for localized prostate cancer.

**Trial registration:**

ClinicalTrials.gov NCT05010343. Retrospectively registered on 18 August 2021

## Background

Radiotherapy is one of the main strategies for localized prostate cancer. The accuracy of irradiation dose delivery to the target volume is closely related to the higher local control as well as the lower side effects, and a higher radiation dose to the target may further increase survival. Results from the dose-escalation photon study [1] indicated that a radiation dose greater than 76Gy could significantly improve the biochemical relapse-free survival in patients with localized prostate cancer and the dose greater than 76Gy or less than 76Gy may achieve different 10-year failure-free survival rates of 73% and 50%, respectively. From the National Comprehensive Cancer Network (NCCN) guidelines [2], the recommended photon irradiation dose for low-risk localized prostate cancer should be 75.6–79.2Gy, and at least 81Gy for intermediate/high-risk localized disease. Thus, increasing irradiation dose might be of great significance for improving the efficacy of prostate cancer.

Currently, localized prostate cancer is mainly irradiated with conventional photon beam. Although intensity-modulated radiation therapy (IMRT)/image-guided radiation therapy (IGRT) could get better dose conformity than 3-dimensional conformal radiation therapy (3DCRT), the irradiation dose was still limited due to the surrounding dose-limiting structures such as rectum and bladder; otherwise, it might lead to severe gastrointestinal (GI) and genitourinary (GU) toxicities, which affect the patients’ quality of life. In addition, from the perspective of fractionation dose, conventional fractionated radiotherapy of prostate cancer might not achieve the best therapeutic effect [3–5]. Actually, although long-term results of the M. D. Anderson randomized trial [1] demonstrated that dose-escalation of conventional photon radiotherapy did improve freedom from biochemical and clinical progression, the 10-year incidence of grade 2 or greater GI toxicity was higher in the high-dose arm than in the low-dose arm (26% vs 13%, *p* = 0.013), as was the incidence of grade 2 or greater GU toxicity (13% vs 8%). Similarly, despite improved clinical or biochemical failure-free survival in the Dutch Phase III trial [6], the 7-year rate of late grade 2 or greater GI toxicity was 35% in the 78Gy arm versus 25% in the 68Gy arm (*p* = 0.04). A meta-analysis [7] showed that high-dose radiotherapy (74–80 Gy) was associated with an increased risk (odds ratio 1.72) of late grade 2 or higher GI toxicity compared with conventional-dose radiotherapy (64–70.2 Gy). Consequently, a better radiotherapy strategy is eagerly wanted.

Carbon ion radiotherapy (CIRT) [8] is currently a novel and powerful tool for radiotherapy which could possess a narrow Bragg peak-like depth dose and a sharp gradient at depths near its distal penetration. The CIRT with intensity-modulated carbon-ion therapy (IMCT) delivery provides better dose conformity to the prostate compared to conventional radiotherapy. As a kind of high linear energy transfer (LET) ray, the relative biological effectiveness (RBE) of CIRT is substantially higher than that of photon and proton-based irradiation, and the RBE value is suggested to be 2–3-fold over photon, producing a more powerful tumor cytotoxicity than conventional radiotherapy. Due to its inspiring physical and biological advantages, CIRT has been gradually proceeded to treat several malignancies [9–12], such as head and neck carcinoma, soft tissue sarcoma, osteosarcoma, lung cancer, and liver cancer with reported satisfactory clinical efficacy. Compared with photon therapy, data published by Japan [13–17] recommended hypofractionated CIRT to be one of the best choices for localized prostate carcinoma, especially for intermediate- and high-risk prostate cancer, with improved 5- and 10-year progression-free survival rates and the favorable late GU/GI toxicities. Previous studies [3, 4, 18] also reported the low alpha/beta value of prostate cancer cell, suggesting that prostate cancer might be more suitable for hypofractionated radiotherapy.

For definitive irradiation of localized prostate cancer, the clinical target volume is mainly defined as the entire prostate and part of the seminal vesicle based on the different risk groups, with a uniform dose delivery. Numerous studies [19–21] have shown that either local recurrence or residual lesions mainly occurred in the primary visible site, and may be correlated with biochemical recurrence of prostate cancer after definitive radiotherapy. A meta-analysis [22] further suggested that every 1-Gy increase in irradiation dose may reduce the risk of biochemical relapse by approximately 1.8%. Based on a multicenter result [23] for multiple systemic tumors, the survival rate was significantly improved after an additional 10–20% dose boost to the radio-resistant site. Tomé et al. [24] set up a prognosis model to predict the efficacy of simultaneous integrated boost (SIB) radiation therapy for solid tumors and revealed that if a boost dose ratio of 1.20–1.30 was given to 60–80% of the tumor, the estimated tumor control probability (TCP) values would rise from 50% to 70%. Based on equivalent uniform dose, Kim et al. [25] compared expected local tumor control and normal tissue toxicities between SIB technique targeting high-risk recurrence area and homogeneous dose technique using voxel-based iso-TCP maps. They found that SIB was a more effective method than homogeneous dose technique, significantly improving local control rate without increasing the normal tissue complications. Dosimetric studies [26, 27] and clinical data [28, 29] on volumetric modulated arc therapy (VMAT) and IMRT technologies further proved that SIB technique of prostate cancer may not increase the irradiation dose and toxic reactions of rectal and bladder, which indicated that simultaneous integrated boost irradiation had the advantage of improving the treatment efficacy without increasing the side effects for prostate cancer. Thus, patients might benefit from the SIB technique for solid tumors with improved tumor control and limited normal tissue damage.

This is a randomized controlled phase II clinical trial, whose objective is to integrate SIB technique with carbon ion treatment for patients with localized prostate cancer and further assess its safety and efficacy.

## Methods/design

### Trial organization/coordination

**Fig. 1:**
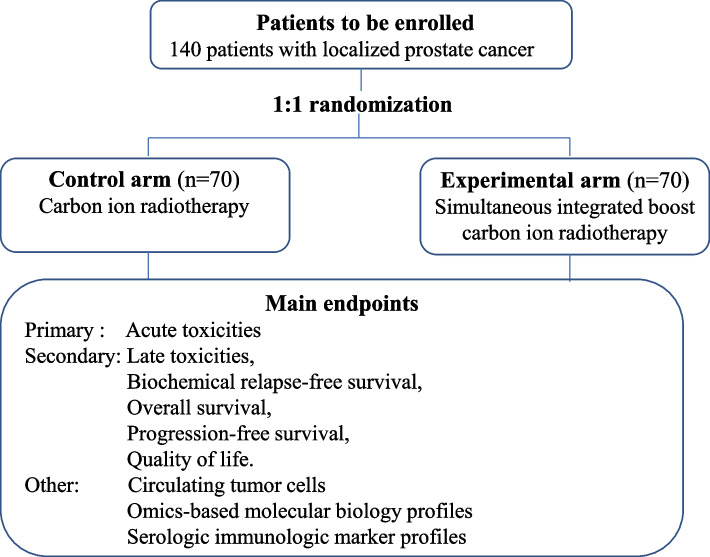
The flow chart of the current trial

This study is designed as an open-label, prospective, single-center, randomized two-armed (carbon ion radiotherapy vs. simultaneous integrated boost radiotherapy) study, primarily evaluating the differences in the severity and incidence of acute toxicity in patients with localized prostate cancer treated with carbon ion radiotherapy and simultaneous integrated boost carbon ion radiotherapy and further optimizing carbon ion therapy for prostate cancer. The rationale for this SIB approach is to provide a greater overall tumor-killing effect to prolong the biochemical recurrence-free survival without additional toxicity as compared with standard treatment. The two study arms are defined by different treatment strategy with arm A (carbon ion radiotherapy at 65.6 Gy in 16 fractions) and arm B (SIB carbon ion radiotherapy to the imaging-visible malignancy at 72Gy in 16 fractions). The flow chart of this phase II trial is illustrated in Fig. 1. The study carries out in Shanghai Proton and Heavy Ion Center (SPHIC) in China, and ethical consent was obtained from the ethics committee of SPHIC before trial initiation. The number of the ethics committee of SPHIC (Institutional Review Board) is 2006-62-03. All patients have written informed consent before inclusion in the trial.

### Patient selection

#### Inclusion criteria

1. Histopathologically confirmed primary prostate cancer
2. Stage cT1-3N0M0 at the time of initial diagnosis, with solid prostate tumor visible on both multiparametric magnetic resonance imaging (mpMRI) and 68GA-PSMA PET/CT imaging
3. Age between 45 and 85 years
4. No regional lymph node metastasis and distant metastasis confirmed by CT, MRI, bone scan, and PET/CT (PSMA)
5. No metal implants such as artificial hip joint in the irradiation field or in the irradiation pathway that can significantly affect the dose distribution
6. ECOG 0 ~ 2; No complications that might affect radiotherapy such as severe pulmonary hypertension, cardiovascular disease, peripheral vascular disease, or serious chronic heart disease; heart function grade 1 (according to the NYHA classification grading of cardiac function)
7. Acceptable hematopoietic function and liver/kidney function:
  - Hematopoietic function: hemoglobin ≥ 90g/L, platelet ≥ 70 × 10^9^/L, white blood cell ≥ 3 × 10^9^/L
  - Liver function: ALT and AST<1.5 times of upper limits of normal (ULN), bilirubin < 1.5 × ULN
  - Renal function: serum creatinine ≤ 140 μmol /L
8. Signed written informed consent

#### Exclusion criteria

1. With other uncontrolled primary malignancies
2. Without pathology diagnosis
3. Pathological types of non-adenocarcinoma of prostate (such as small cell carcinoma, sarcoma, etc.)
4. Lymph nodes or distant metastasis
5. Previous prostatectomy or pelvic radiotherapy
6. Obvious adverse effects related to previous treatment, such as urinary incontinence, hematuria, and bloody stools, which may be aggravated or induced by radiation therapy
7. Active medical implants, e.g., pacemaker and defibrillator, which may be interfered with the normal function by high-energy radiation or may affect the dose in the target area
8. Drug abuse or alcohol dependence
9. HIV positive, including previous antiretroviral treatment; syphilis active stage; chronic hepatitis B, virus replication period; active stage of hepatitis C
10. A history of mental illness, which may hinder the completion of treatment
11. Poor general health, i.e., KPS<70 or ECOG>2
12. With serious complications that may interfere with radiotherapy, including:
  - Unstable angina pectoris, congestive heart failure, or myocardial infarction requiring hospitalization in the past 6 months
  - Acute bacterial or fungal infection
  - Exacerbation of chronic obstructive pulmonary disease or other respiratory diseases requiring hospitalization
  - Inflammatory bowel disease or connective tissue disease, such as active scleroderma and lupus (contraindicated by radiotherapy)
  - Liver dysfunction (ALT and AST≥ 1.5 × ULN, bilirubin ≥ 1.5 × ULN)
13. Immunosuppressed patient
14. The presence of other disorders or other factors that may affect proton or carbon ion therapy
15. Without civil capacity or with limited civil capacity

### Patient assessments

When patients meet the trial conditions, they are provided with relevant information including potential risks and benefits of participating in the trial. Patients may be enrolled and randomized by the principal investigator in this trial once written consent has been obtained and all required documentation will be provided by SPHIC. Patients will be randomly assigned to either arm A or arm B in a 1:1 ratio using computer-generated random numbers by the principal investigator, and sequentially numbered after being randomly assigned. Before radiation, a detailed medical history inquiry and physical examination are required. Pathological diagnosis of the primary disease, including pathological classification, Gleason score, and positive puncture rate, is necessary before treatment. Also, patients need laboratory tests, such as peripheral blood routine, serum chemistry, urine and stool routine examination, the levels of prostate-specific antigen (PSA), fPSA, testosterone, neuron-specific enolase (NSE), and tests for HIV, syphilis, hepatitis. In addition, CT of the chest, mpMRI of the prostate, and B-scan ultrasonography of upper abdomen and inguinal and supraclavicular lymph nodes are conducted before irradiation. Bone scan and 68Ga-PSMA PET/CT are performed to comprehensively evaluate the lesion range and staging. Cardiac function examinations including electrocardiogram are taken and cardiac ultrasound, 24-h Holter examination, and coronary angiography are conducted if necessary. Moreover, the effects of carbon ion therapy on quality of life are evaluated by the five-dimensional European quality of health scale (EQ-5D), International Prostate Symptom Score (IPSS), and Expanded Prostate Cancer Index Composite (EPIC) questionnaires.

During irradiation, all patients are regularly assessed for acute toxicities of normal tissues at least once a week until they have subsided. The adverse events, grading, and correlation with radiotherapy are documented at each evaluation. Specific evaluation contents include weekly routine blood tests to observe the hematological toxicity, monthly liver function tests to observe the liver toxicity, weekly routine urine examination to observe the occurrence of hematuria and urinary tract infection, IPSS questionnaire to evaluate urinary system toxicity, and weekly routine stool examinations and occult blood examinations combined with the symptoms of intestinal toxicity to exclude radioactive enteritis. Patients are treated with combined endocrine therapy, either neoadjuvant endocrine therapy or concurrent endocrine therapy, depending on their grading. When necessary, a supportive medication is initiated or adapted. When patients experience treatment-related adverse events, appropriate medications will be given according to the severity of the disease. If patients have an unexpected injury during the clinical study, financial compensation will be made in accordance with Chinese laws and regulations. Additionally, quality of life is collected during and at the end of radiotherapy. Table 1 summarizes the schedule.

**Table 1:** Schedule of the trial

	Before radiation	During radiation	At the end of radiation (the final day of radiation)	Follow-up after treatment						
		Every week		The first year				The second and third years	The fourth year	From the fifth year
				Weeks 4–6 (first time)	The 3rd month	The 6th month	The 12th month	Every 3 months	Every 6 months	Every year
**Written informed consent**	**√**									
**Medical history/physical examination**	**√**	**√**	**√**	**√**	**√**	**√**	**√**	**√**	**√**	**√**
**KPS, ECOG**	**√**	**√**	**√**	**√**	**√**	**√**	**√**	**√**	**√**	**√**
**Pathological diagnosis**	**√**									
**Inclusion and exclusion criteria**	**√**									
**Peripheral blood routine examination**	**√**	**√**^**a**^	**√**	**√**	**√**	**√**	**√**	**√**	**√**	**√**
**Serum chemistry**	**√**	**√**^**a**^	**√**	**√**	**√**	**√**	**√**	**√**	**√**	**√**
**Urine and stool routine examination**	**√**	**√**^**a**^	**√**	**√**^**a**^	**√**^**a**^	**√**^**a**^	**√**^**a**^	**√**^**a**^	**√**^**a**^	**√**^**a**^
**Levels of PSA, fPSA, testosterone, and NSE**	**√**		**√**	**√**	**√**	**√**	**√**	**√**	**√**	**√**
**Tests for HIV, syphilis**	**√**									
**Tests for HBV, HCV**	**√**									
**Cardiac function examination**	**√**									
**CT of the chest**	**√**						**√**	**Annually**	**Annually**	**Annually**
**CT scan or ultrasound of the abdomen**	**√**		**√**	**√**	**√**	**√**	**√**	**√**	**√**	**√**
**mpMRI of prostate**	**√**		**√**	**√**	**√**	**√**	**√**	**Annually**	**Annually**	**Annually**
**Bone scan examination**	**√**^**a**^						**√**	**Annually**	**Annually**	**Annually**
**68Ga-PSMA PET/CT**	**√**^**b**^		**√**^**b**^				**√**	**Annually**	**Annually**	**Annually**
**Evaluation of acute toxicities (NCI-CTACE)**	**√**	**√**	**√**	**√**	**√**			**√**		
**Evaluation of late toxicities (RTOG/ EORTC)**					**√**	**√**	**√**	**√**	**√**	**√**
**EPIC**	**√**	**√**	**√**		**√**	**√**	**√**	**Annually**	**Annually**	**Annually**
**EQ-5D**	**√**	**√**	**√**		**√**	**√**	**√**	**Annually**	**Annually**	**Annually**
**IPSS**	**√**	**√**	**√**		**√**	**√**	**√**	**Annually**	**Annually**	**Annually**

^a^when necessary

^b^recommended

All the patients will be followed up according to the protocol every 3 months in the initial 3 years after the completion of treatment, every 6 months for an additional year, and every year thereafter. Follow-up examinations include complete history, physical examination, digital rectal examination, PSA, complete blood count, serum chemistry, CT of the chest, CT scan or ultrasound of the abdomen, mpMRI of the prostate, bone scan examination, and 68Ga-PSMA PET/CT which will be performed earlier if recurrence is suspected. Every patient is recommended to evaluate PSA at the same laboratory. In addition, quality of life should be assessed regularly during follow-up.

### Radiation therapy

#### Definition of target volumes and organs at risk

According to the risk factors of prostate cancer, there are three different definitions of clinical target volume (CTV). For the low-risk group, CTV is defined as the entire prostate; for the intermediate-risk group, CTV is defined as the entire prostate gland and inferior 1-1.5cm of seminal vesicles; and for high-risk and very high-risk groups, CTV is defined as the entire prostate gland and inferior 2–2.5cm of seminal vesicles.

The determination of the target boost area of prostate cancer is the key to the implementation of SIB technology. Based on the European Association of Urology (EAU) [30], the NCCN [2], and the European Society of Urogenital Radiology (ESUR) guidelines [31], mpMRI is used for the imaging diagnosis of prostate cancer due to its ability of great soft tissue resolution and the accuracy to distinguish between prostate tumors and normal prostate tissues [32]. A meta-analysis [33] of 29 prospective studies also showed that the sensitivity and specificity of mpMRI for the diagnosis of prostate cancer were 0.87 and 0.68, respectively. In addition, as a type II transmembrane protein, prostate-specific membrane antigen (PSMA) can be highly expressed in prostate cells, and its expression level is positively correlated with the stage and the grade of prostate cancer. 68Ga-PSMA PET/CT is assigned as an emerging diagnostic technique for prostate cancer based on PSMA molecular imaging. The positive diagnosis rate of prostate cancer can reach 69%, with 80% diagnostic sensitivity and 97% specificity, even 42% at very low PSA levels (<0.2 ng/ml) [34], and 68Ga-PSMA PET/CT has been gradually adopted clinically to guide radiotherapy planning for prostate cancer [35, 36]. Therefore, combined 68Ga-PSMA PET/CT and mpMRI might accurately locate the target area of prostate tumors, and CTV boost of the SIB group is defined as solid prostate tumors that are visible on mpMRI and 68Ga-PSMA PET/CT images in this trial.

The planning target volume (PTV) is defined as the CTV plus 8 mm in lateral (beam direction) and 5 mm anterior-posterior as well as in the inferior-superior margin.

#### Definition of the dosage

For arm A, the total dose for CTV should be 65.6GyE in 16 fractions (4.1GyE per fraction) in 4 weeks. For arm B, the total dose for CTV should also be 65.6GyE in 16 fractions (4.1GyE per fraction) and CTV boost be 72GyE in 16 fractions (4.5GyE per fraction) simultaneously. Moreover, 95% of the prescription dose should cover 100% of the CTV and 90% of the prescription dose should cover 100% of the PTV in both arms. The dose constraints of organs at risk are defined as rectum Dmax ≤ 100%PD, V60 ≤ 3cc, V55 ≤ 7cc, V50 ≤ 10cc and V30 ≤ 15cc, bladder Dmax ≤ 100%PD, D15% ≤ 40GyE, and D5% ≤ 50GyE.

### Duration of the study

The primary endpoint is to compare the treatment-induced grade 2 or greater acute toxicity between arm A and arm B. Seventy patients are planned to be enrolled in each arm, and 140 patients are enrolled in total.

It will take about 5 years to complete the enrollment of all patients and every patient should complete at least 3 months of follow-up work after radiotherapy. The study is expected to start in October 2020 and primarily be completed in July 2025.

### Outcome measures

The primary objective of this phase II randomized study is to evaluate the differences of acute toxicities in patients with localized prostate cancer treated with carbon ion radiotherapy and SIB carbon ion radiotherapy.

Late toxicities, biochemical relapse-free survival, overall survival, and progression-free survival are the secondary outcome measures to preliminarily explore the optimal dose and efficacy of SIB carbon ion radiotherapy of localized prostate cancer.

Additionally, 25ml of peripheral blood and 35ml of urine will be collected prospectively before and after treatment for the detection of immune and circulating tumor cells related to prostate cancer carbon ion therapy. The purpose of these indicators’ detection is to evaluate the efficacy and toxicity of prostate cancer carbon ion therapy and then to predict the prognosis of the disease. Before the collection of biological specimens, an informed consent form will be provided by the principal investigator to patients.

Moreover, EQ-5D, IPSS, and EPIC questionnaires are used to evaluate the effect of carbon ion radiotherapy on patients’ quality of life. IPSS would be used to assess the lower urinary tract symptoms. EPIC would be used to evaluate the urinary, bowel, and sexual symptoms.

### Evaluation of side effects

Acute toxicities from treatment are graded according to NCI-CTCAE version 4.03 [37]. Late toxicities from treatment are graded according to Radiation Therapy Oncology Group/European Organization for Research and Treatment of Cancer (RTOG/EORTC) criteria [38]. Late toxicities are defined as symptoms first occurring or lasting > 90 days from radiotherapy.

### Criteria for discontinuation

Patients should be discontinued from the study if any of the following conditions occur:

1. Occurrence of treatment-related serious adverse events.
2. Patients’ requirement to withdraw from the study.
3. Disease progression.
4. Serious breach of the research protocol.
5. Death of the subject.
6. The investigator considers that the patient should be discontinued from the study.

### Plans to promote participant retention and complete follow-up

The subjects will be recruited once they are hospitalized and they will remain in the site until discharge. Patients have the right to withdraw during the study, which will not affect the subsequent medical treatment. If the subject withdraws, the investigator must record the reason and date of withdrawal on the subject’s case report form (CRF). All subjects who withdrew due to adverse events or abnormal laboratory test results must be followed up until they recover or stabilize, and subsequent outcomes recorded. If patients develop disease progression, they should be followed up until death. If patients fail to receive all treatment regimens for any reason other than disease progression, follow-up including efficacy, toxicity, and quality of life should still be performed.

### Data monitoring/auditing

The study investigators will monitor the integrity of study data and participant safety. During the period of recruitment, regular analyses will be provided to the Institutional Review Board, who will give advice on the study conduction, protocol modification, and trial halt if needed.

Interim analyses were performed in order to assess the safety and efficacy of treatment. If a significant lack of efficacy or an unacceptable risk-benefit balance is found, the Institutional Review Board will organize an expert group to review this situation and make the final decision to terminate the trial.

The trial will be regularly audited on-site and online by experts, independent from investigators.

### Protocol amendments

Any important modifications to the protocol, such as changes to eligibility criteria, outcomes, and analyses will require a formal amendment to the protocol. Such amendment must be submitted to the Institutional Review Board for approval before implementation.

### Statistical analysis

The primary endpoint of the study is to observe the difference in acute toxicity in patients with localized prostate cancer who receive carbon ion radiotherapy and SIB carbon ion radiotherapy. All the patients will be randomly assigned to arm A and arm B in a ratio of 1:1. Assuming a type I error of 0.025 and type II error of 0.2, and under the assumption that the probability of no more than 2 degrees of acute GU and GI toxicities in arm A is 90% (Pc), and the probability of no more than 2 degrees of acute GU and GI toxicities in arm B is also 90% (Pt), with a non-inferiority margin of 0.15, we estimate that a sample size of 63 participants per arm will yield 80% power to show non-inferiority. However, anticipating a possible 10% dropout rate in each group, per arm *n* = 70 patients will be recruited into the study such that a total of *N* = 140 patients is required.

Statistical analysis will be performed using SPSS software (V20.0, SPSS Inc., Chicago, IL, USA). The Mann-Whitney *U* test will be used to compare the differences in acute and late toxicity between the two arms. The Kaplan-Meier method will be used to estimate the biochemical relapse-free survival, progression-free survival, and overall survival of the entire cohort. The association between each of the candidate prognostic factors with biochemical relapse-free survival, progression-free survival, and overall survival rates will be tested using the log-rank test. Multivariate analysis will be performed using the Cox regression model.

Analysis is planned on an intention-to-treat population (ITT), with all participants to be analyzed in the group to which they were randomized. We will make efforts to collect complete data for all participants and minimize missing data. If required, the missing data will be dealt with last observation carried forward method or multiple imputation.

### Patient and public involvement

Patients or the general public will not be involved in the formulation of research questions and study design. Before the study, we will inform patients of the background, plan, possible risks and benefits, and other relevant information of the study. During the study, feedback comments obtained from patients will be taken into full consideration. Moreover, the results of the study will be disseminated to participants mainly by telephone.

## Discussion

Radiotherapy is one of the treatment options for localized prostate cancer, but standard radiation doses (64 to 70 Gy) are not always as effective as previously believed. One solution to improve the efficacy of radiotherapy is increasing the irradiation dose. There is convincing evidence [39–43] that irradiation dose is positively correlated with efficacy. However, further increase in dose to the entire prostate is limited by the tolerance of adjacent normal tissues and may result in higher toxicity rates [1, 42]. Besides, from a radiobiological point of view, α/β ratio of prostate cancer appears to be as low as 1.5 Gy [3, 4, 18], which is even lower than that of the surrounding organs at risk such as the rectum, suggesting that prostate cancer might show some resistance to conventionally fractionated photon radiotherapy. Therefore, there is an urgent need for more effective treatments that can improve therapeutic effects without increasing adverse effects.

As an alternative to external beam radiotherapy, brachytherapy has been integrated as a boost technique or definitive technique in multimodality approaches in the treatment of prostate cancer. Brachytherapy allows very high doses to be delivered inside the prostate, with a sharp dose gradient outside the prostate establishing high conformity to the target volume, thus showing high effectiveness and relatively low morbidity [44]. As a form of brachytherapy that high-activity radiation sources are temporarily placed within the prostate, typically over two to three fractions, high-dose rate brachytherapy (HDRBT) translates dosimetric superiority into excellent clinical results [45, 46]. Radiobiologically, HDRBT also takes advantage of the low α/β ratio of prostate cancer. However, the precise dose delivery is often limited by the technique uncertainty, such as difficulties in accurate needle placement at a desired target in the prostate and the heterogeneity of the prescribed doses. CIRT is one of the strategies that can be employed to deliver a higher biological effect to the prostate.

It is noteworthy that CIRT seems to be beneficial to improve local control and reduce toxicity to normal tissues because of its biophysical advantages. Some centers [14, 15, 17, 47, 48] have conducted several studies of CIRT for prostate cancer, showing the advantages of improved efficacy and reduced side effects. CIRT displays a low dose deposition along the entry channel of the beam and a steep dose deposition in the Bragg peak region and can be concentrated on the CTV. It is also said that CIRT causes less chromosomal damage on normal tissues than photon irradiation does [49]. On top of that, with both high-LET and RBE properties, CIRT causes a larger proportion of tumor cells to be killed through several mechanisms, such as generating more DNA double-strand breaks and inducing mitotic catastrophe, which is concluded by ceramide-dependent-apoptotic cell death [50, 51]. Unsurprisingly, under that circumstance, more tumor-specific antigens are released; thus, stronger local and abscopal anti-tumor immunity is induced [52]. Compared with X-rays, carbon ions increased the exposure of high mobility group box 1 (HMGB1) [53] while relatively reduced the exposure levels of immunosuppressive factors IL-10 and TGF-β in lung cancer cell lines. Additionally, Chiblak et al. [54] found that CIRT prolonged survival of mice, which was attributed to the reduction of M2-like macrophages and MDSCs, increase of CD8+ T cells, and generation of an immunopermissive niche, in contrast to photon radiotherapy. Therefore, CIRT has the potential to induce higher immunogenicity than photons. In spite of carbon ion radiotherapy being a talented modality for malignant tumor patients, the radiation damage to normal tissues adjacent to the tumor still limits therapeutic gain and the optimal irradiation dose is also under investigation.

It is considered that a quarter of prostate cancer patients who underwent definitive external beam radiotherapy may experience recurrence after treatment, and the most common local recurrence site of prostate cancer is the primary macroscopic tumor [19, 55]. In that case, a number of studies [29, 56–58] have explored the effectiveness and safety of the focal boosting strategy of photon radiotherapy. The phase III FLAME [56] trial investigated the benefit of an ablative microboost to the macroscopic visible tumor in 571 patients with intermediate- and high-risk prostate cancer, delivering a simultaneous integrated focal boost to the intraprostatic lesion of 95Gy in 35 fractions with whole prostate gland doses of 77 Gy in 35 fractions. Biochemical progression-free survival was significantly improved in the focal boost arm compared with the standard arm. However, the cumulative incidence of late GU and GI toxicity grade ≥ 2 was 23% and 12% in the standard arm versus 28% and 13% in the focal boost arm, respectively, showing that the differences in toxicities between the two arms were not significant [59]. Therefore, selective dose escalation to the macroscopic tumor instead of the whole prostate gland could be a promising strategy to increase efficacy, while the dose constraints to the rectum and bladder can be maintained [60].

In radiobiology, the α/β ratio is used to quantify the fractionation sensitivity of both normal tissues and tumors. Tissues with lower α/β ratios demonstrate relatively greater fractionation sensitivity, then a therapeutic advantage can be gained by using fewer and larger fractions, namely hypofractionation. This hypofractionation scheme has been validated in the treatment of early breast cancer [61, 62], and in the palliative treatment of lung cancer [63]. As mentioned above, the α/β ratio for prostate cancer is lower than that of the nearby normal tissues, suggesting that prostate cancer may be more suitable for hypofractionation. A number of randomized controlled trials [64–66] showed that moderately hypofractionated photon radiotherapy had similar biochemical control compared to conventionally fractionated photon radiotherapy without increasing late toxicity and this effect seemed to hold true for all baseline clinical risk groups. Several phase III randomized trials [67, 68] comparing ultra-hypofractionated or stereotactic body radiotherapy (SBRT) with conventionally fractionated or moderately hypofractionated radiotherapy showed very promising failure-free survival and favorable acute and late treatment-related toxicities when using SBRT in predominantly low- and intermediate-risk prostate cancer. In addition to photon therapy, hypofractionated CIRT was proven by Japan [14, 17] to yield a good therapeutic outcome and low toxicity rates. Other potential advantages of hypofractionation include decreased overall treatment time, reduced cost, and increased convenience and participant capacity [69].

Thus, the conjunction of focal tumor boosting and hypofractionated regimen [70, 71] could combine the above potential advantages of both strategies. For instance, the multicenter phase II hypo-FLAME study [71], enrolling 100 patients with intermediate or high-risk prostate cancer, proved that extreme hypofractionated doses of 35 Gy in 5 weekly fractions to the entire prostate gland with a simultaneous ablative microboost up to 50 Gy to the mpMRI-visible tumor was effective and safe.

Previously, we conducted a phase I/II dose-escalation CIRT study for localized prostate cancer, and the results showed that the irradiation dose of 65.6GyE/16 fractions to the whole prostate and seminal vesical was safe. Furthermore, dosimetric studies [26, 27] and clinical data [28, 29] proved that SIB irradiation had the advantage of improving the treatment efficacy for prostate cancer without increasing irradiation dose and toxicities of rectal and bladder. Therefore, we think it is reasonable to treat localized prostate cancer using SIB carbon ion radiotherapy technique, and this first randomized controlled trial of SIB carbon ion radiotherapy for localized prostate cancer is designed.

In summary, this study adopts 68Ga-PSMA PET/CT and mpMRI to locate target area of the visible prostate cancer, and SIB technology of CIRT to perform precise radical radiotherapy for localized prostate cancer. Through observing treatment-related toxicities after treatment; investigating the biochemical relapse-free survival, overall survival, and progression-free survival; and assessing quality of life, efficacy and safety of SIB carbon ion radiotherapy for localized prostate cancer can be preliminarily explored and optimal carbon ion treatment mode for localized prostate cancer can be hopefully determined.

## Dissemination of study results

The results of the trial will be presented at national and international scientific conferences and submitted for publication in peer-reviewed journals.

The investigators will consider all proposed publications, with the final decision on content and authorship resting with the Principal Investigator. The role of each author will be published in line with journal requirements.

Once the trial has ended and the data has been collected, analyzed, and published, the anonymous database, the full protocol, statistical code, and other materials will be available to other researchers upon request to the principal investigator.

## Trial status

The trial protocol is Version 2.0, 13 August 2020. Recruitment began on 15 October 2020 and is anticipated to be completed by 1 July 2025.

## Data collection and management

CRFs will be recorded by investigators and the research coordinator. CRFs will be stored and archived by investigators.

There are special personnel for regular follow-up and data entry, and the study site has a special database for data storage. All study-related information will be stored securely at the study site.

## Confidentiality

All study-related information, including all laboratory specimens, reports, data collection, process, and administration forms will be stored securely at the study site, identified by a coded ID number to ensure confidentiality of participants. Only authorized personnel have access to patient information and conduct research.

## Data Availability

Data sharing is not applicable to this article as no datasets were yet generated or analyzed during this ongoing study.

## Abbreviations

3DCRT: 3-dimensional conformal radiation therapy
ALT: Alanine transaminase
AST: Aspartate transaminase
CIRT: Carbon ion radiotherapy
CTV: Clinical target volume
EAU: European Association of Urology
ECOG: Eastern Cooperative Oncology Group
EPIC: Expanded Prostate Cancer Index Composite
EQ-5D: Five-dimensional European quality of health scale
ESUR: European Society of Urogenital Radiology
GI: Gastrointestinal
GU: Genitourinary
HMGB1: High mobility group box 1
IGRT: Image-guided radiation therapy
IMCT: Intensity-modulated carbon-ion therapy
IMRT: Intensity-modulated radiation therapy
IPSS: International Prostate Symptom Score
KPS: Karnofsky Performance Status
LET: Linear energy transfer
MDSC: Myeloid-derived suppressor cell
mpMRI: Multiparametric magnetic resonance imaging
NCCN: National Comprehensive Cancer Network
NCI-CTCAE: National Cancer Institute Common Terminology Criteria for Adverse Events
NSE: Neuron-specific enolase
NYHA: New York Heart Association
PET/CT: Positron emission tomography/computed tomography
PSMA: Prostate-specific membrane antigen
PTV: Planning target volume
RBE: The relative biological effectiveness
RTOG/EORTC: Radiation Therapy Oncology Group/European Organization for Research and Treatment of Cancer
SBRT: Stereotactic body radiotherapy
SIB: Simultaneous integrated boost
SPHIC: Shanghai Proton and Heavy Ion Center
TCP: Tumor control probability
ULN: Upper limits of normal
VMAT: Volumetric modulated arc therapy
